# Antibiotics Increase Gut Metabolism and Antioxidant Proteins and Decrease Acute Phase Response and Necrotizing Enterocolitis in Preterm Neonates

**DOI:** 10.1371/journal.pone.0044929

**Published:** 2012-09-13

**Authors:** Pingping Jiang, Michael Ladegaard Jensen, Malene Skovsted Cilieborg, Thomas Thymann, Jennifer Man-Fan Wan, Wai-Hung Sit, George L. Tipoe, Per Torp Sangild

**Affiliations:** 1 School of Biological Sciences, The University of Hong Kong, Hong Kong, People’s Republic of China; 2 Department of Anatomy, The University of Hong Kong, Hong Kong, People’s Republic of China; 3 Department of Human Nutrition, University of Copenhagen, Frederiksberg, Denmark; Emory University School of Medicine, United States of America

## Abstract

**Background:**

The appropriate use of antibiotics for preterm infants, which are highly susceptible to develop necrotizing enterocolitis (NEC), is not clear. While antibiotic therapy is commonly used in neonates with NEC symptoms and sepsis, it remains unknown how antibiotics may affect the intestine and NEC sensitivity. We hypothesized that broad-spectrum antibiotics, given immediately after preterm birth, would reduce NEC sensitivity and support intestinal protective mechanisms.

**Methodology/Principal Findings:**

Preterm pigs were treated with antibiotics for 5 d (oral and systemic doses of gentamycin, ampicillin and metrodinazole; AB group) and compared with untreated pigs. Only the untreated pigs showed evidence of NEC lesions and reduced digestive function, as indicated by lowered villus height and activity of brush border enzymes. In addition, 53 intestinal and 22 plasma proteins differed in expression between AB and untreated pigs. AB treatment increased the abundance of intestinal proteins related to carbohydrate and protein metabolism, actin filaments, iron homeostasis and antioxidants. Further, heat shock proteins and the complement system were affected suggesting that all these proteins were involved in the colonization-dependent early onset of NEC. In plasma, acute phase proteins (haptoglobin, complement proteins) decreased, while albumin, cleaved C3, ficolin and transferrin increased.

**Conclusions/Significance:**

Depressed bacterial colonization following AB treatment increases mucosal integrity and reduces bacteria-associated inflammatory responses in preterm neonates. The plasma proteins C3, ficolin, and transferrin are potential biomarkers of the colonization-dependent NEC progression in preterm neonates.

## Introduction

Intestinal microbiota, prematurity and inappropriate enteral feeding are regarded as three key risk factors for the onset and progression of necrotizing enterocolitis (NEC), a serious intestinal inflammatory disease in preterm infants with high morbidity and mortality [1]. The crucial role of intestinal microbiota has been documented in many studies, including in our own studies on germ-free, fetal or postnatal immature pigs that are protected against NEC [2]. The intestinal microbiota in preterm neonates is less diverse than in term neonates, especially when delivered by caesarean section [2], and this may predispose the gut to pathogenic *E. coli, Clostridium, Klebsiella* and *Bacterioides* species [2]–[4]. Overgrowth of these pathogenic species triggers inappropriate inflammatory processes in the immature intestine which sensitize to further translocation of pathogenic bacteria and toxins, leading to sepsis and necrosis. Although the intestinal commensal microbiota plays a crucial role in the onset and progression of NEC, no single causative pathogenic microbial species has been identified [5].

Antibiotic regimens in clinical neonatology are highly variable and empirical and mainly used with the aim to prevent and treat systemic sepsis. Antibiotics such as ampicillin plus cefotaxime or aminoglycoside, clindamycin and/or metronidazole have been recommended for NEC treatment in the USA [6], [7], while penicillin, gentamicin and metronidazole are sometimes used in the UK [8]. Molecular profiling of infant fecal microbial communities after such antibiotics treatment shows dramatic reductions in the total bacterial densities and alterations in population composition [6].

The widespread use of broad-spectrum antibiotics in clinical neonatology has some obvious disadvantages. These include possible development of antibiotics-resistant microbes and the potential to induce an inappropriate delay in bacterial colonization and a microbiota composition that may predispose to NEC [4]. Despite these reservations, we believe that the widespread therapeutic use of antibiotics in virtually all neonatology units justifies a renewed look at the possible benefits of a more controlled, prophylactic antibiotic treatment that delays gut microbial colonization. It is noteworthy that oral treatment with vancomycin or gentamycin has been associated with decreased incidence of NEC [9].

Studies in appropriate animal models of preterm birth and NEC may offer an opportunity to re-think the optimal time and mode of antibiotics treatment for newborn preterm infants. A high proportion of preterm pigs spontaneously develop diet- and colonization-dependent NEC lesions that are similar to those infants [1], [10]._ENREF_10 We hypothesized that immediate postnatal treatment of preterm pigs with broad-spectrum antibiotics would reduce the overall gut bacterial density and prevent bacterial-induced deficits in cellular proteins important for early NEC progression. In our previous studies, we adopted gel-based proteomics to detect the expression change of hundreds of different proteins in response to feeding, bacterial colonization and NEC [11], [12]. These studies served as the background to evaluate the specific effect of antibiotics on both the intestinal and plasma proteome in formula-fed preterm pigs. Antibiotics were administered both orally and parenterally to ensure that a rapid antimicrobial effect in both the gut and the systemic circulation. We document how antibiotics affect intestinal proteins important for intestinal structure, function and immunity. A concomitant analysis of the plasma protein proteome allowed us to suggest new candidates for circulating biomarkers of the colonization-dependent NEC development.

## Results

### NEC, Mucosal Morphology, Brush-border Enzymes and Microbiology

**Figure 1:**
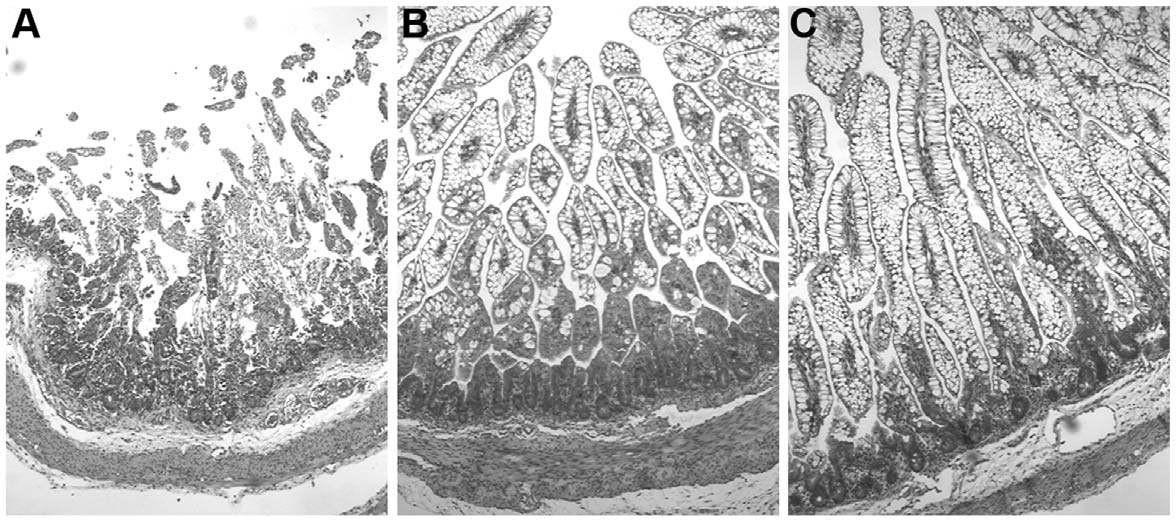
Representative micrographs of the small intestine cross sections. *Panel A:* untreated, NEC score  =  3. *Panel B:* untreated, NEC score  =  2. *Panel C:* AB, NEC score  =  1.

At the time of euthanasia on day 5, the untreated piglets showed variable degrees of initial NEC symptoms such as general weakness, diarrhea and discoloration, whereas the AB pigs all appeared normal. In this study, we deliberately aimed to analyze the intestine of pigs prior to the time when they would become severely affected with NEC pathological lesions, as this would make the interpretation of the proteomic data less meaningful and indicate intestinal proteome of NEC pathology rather than initial events of NEC progression. According to our macroscopic NEC criteria, untreated pigs showed higher NEC incidence than AB pigs (6/6 vs. 0/6, p<0.05) and higher tissue NEC score specifically in the middle intestine (median 2 vs. 1, Mann-Whitney test, p<0.05). Representative hematoxylin and eosin (HE) stained histological slides of the middle small intestine in the AB and the untreated pigs are shown in **Figure 1**. The healthier state of the AB intestines was indicated by higher villi, absence of hemorrhage and no separation of different layers (**Figure 1C**), whereas, the untreated pigs showed various features related to NEC in different individual pig from villous atrophy, hemorrhage (**Figure 1A**) to separation of mucosa layers (**Figure 1B**).

**Figure 2:**
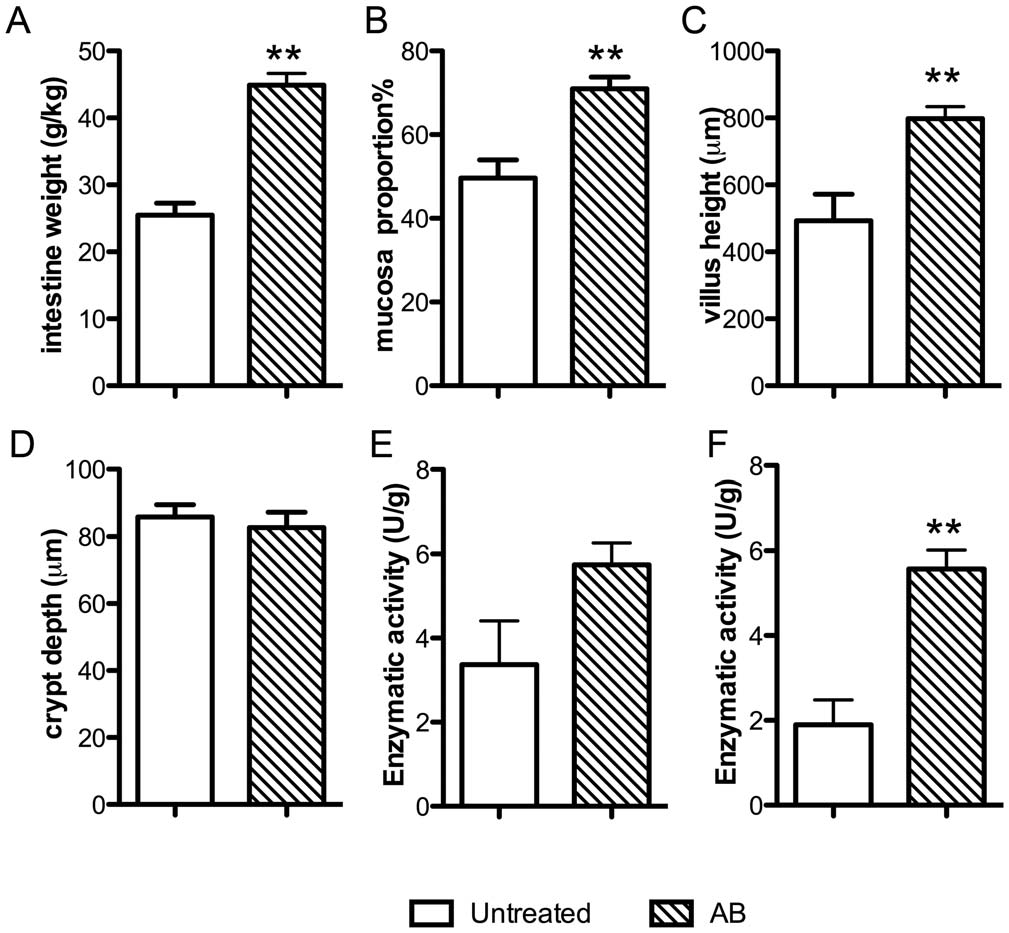
Small intestine weight, mucosa weight, villus height, crypt depth and enzymatic activities of ApN and ApA. Data are presented as mean±SEM. ** p<0.01 in AB *vs* untreated pigs.

Small intestine weight (g) over body weight (kg) (**Figure 2A**), mucosa portion of intestinal segments (**Figure 2B**), villus height (**Figure 2C**) were significantly higher in AB pigs (p<0.01), whereas there was no significant difference in crypt depth between AB and untreated pigs (**Figure 2D**). Activities of lactase (p = 0.11), aminopeptidase N (ApN, p = 0.08, **Figure 2E**) and aminopeptidase A (ApA, p<0.01, **Figure 2F**) were higher, while maltase (p = 0.06) and dipeptidylpeptidase IV (DPPIV) activities were lowered in AB pigs. Significantly lower bacterial number (p<0.001) was observed in the caecum contents of AB pigs compared with untreated pigs, both aerobes (0.3±0.1 vs. 7500±1400, ×10^6^) and anaerobes (0.3±0.1 vs. 7600±1400 ×10^6^) using calf blood agar culturing. All the bacteria found in the cultures were gram-positive species.

### Intestinal Proteomics

**Figure 3:**
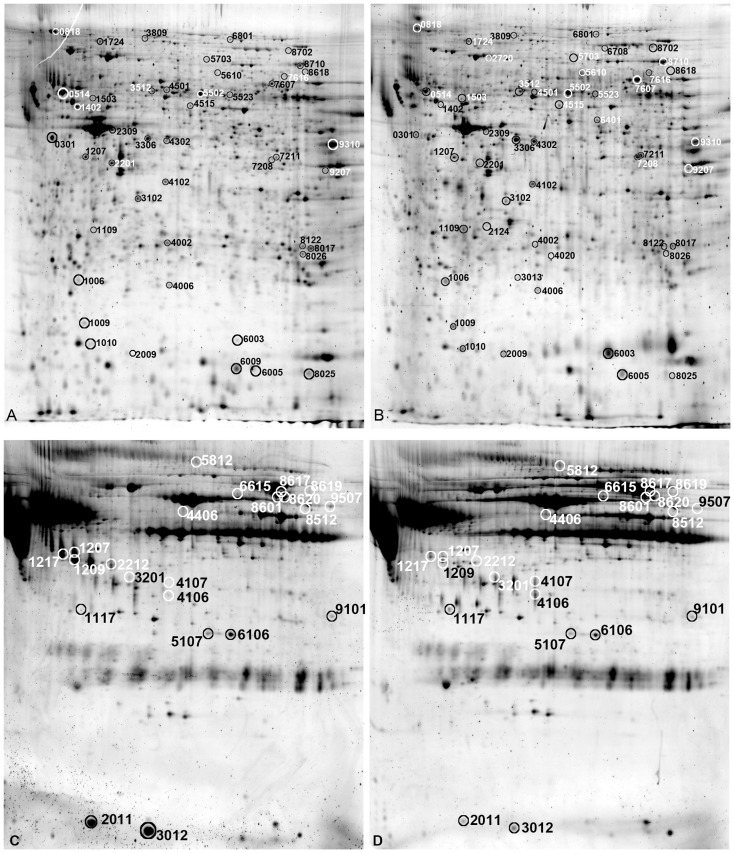
2-DE proteome graphs of the mid small intestine and blood plasma. ***Panel*** ** ***A*** *:* small intestine, untreated; ***Panel B***, small intestine, AB; ***Panel C***, plasma, untreated; ***Panel D***, plasma, AB. Spot number was assigned by the analysis software and correlated with the ones presented in **Tables 1** and **2**.

**Figure 3A and B** shows representative intestinal proteomes of the AB and the untreated pigs. There were 53 differentially expressed protein spots that were successfully identified. **Table 1** shows a descriptive summary of the identified proteins, including spot numbers (circled in **Figure 3A, B**), protein name, GenInfo identifier, expression quantity, expression change the identified proteins. The spots were classified into 13 groups according to their major physiological functions, as related to heat shock proteins, pathogen response, antioxidation, complement system, protein synthesis, processing and degradation, carbohydrate metabolism, mRNA metabolism, amino acid metabolism, fatty acid metabolism, pyrimidine metabolism, iron homeostasis, intracellular traffic, ion channel, cytoskeleton and cell mobility, and secretory proteins. The biological functions of the identified proteins and their proposed role in mediating the physiological and clinical effects of AB treatment are discussed in the Discussion section.

**Table 1:** Intestinal proteins identified with differential expression between the AB and the untreated pigs (p<0.05).

Spotno^a^	Protein Name	GI Id^b^	Protein Score^c^	Expression Level^d^ (AB)	Expression Level^d^ (Untreated)	Fold Change (AB/Untreated)
***Heat shock proteins***						
0818	heat shock protein 90B1	gi|27807263	186	25.8±6.2	80.0±15.1	–3.1
2720	heat shock protein 70A2	gi|296439628	111	9.9±1.4	0	+
4002	heat shock protein B1	gi|55926209	198	22.2±3.9	74.7±15.5	–3.3
4020	heat shock protein B1	gi|50916342	207	42.5±10.9	0	+
***Pathogen response***						
6009	regenerating islet-derived protein 3γ	gi|221325622	153	0	113.6±32.0	–
***Antioxidation***						
7607	catalase	gi|50979303	479	93.2±11.7	46.8±5.8	+2.0
***Complement system***						
8618	complement C3	gi|47522844	76	4.1±1.0	7.8±0.9	–1.9
***Protein synthesis, processing and degradation***						
0301	laminin receptor precursor	gi|161761214	325	33.7±5.3	92.8±11.4	–2.8
1009	elongation factor 1- γ	gi|74001912	171	117.4±9.3	31.8±5.8	+3.7
1402	protein disulfide isomerase-related protein 5	gi|1710248	352	10.9±1.9	37.2±6.8	–3.4
2009	eIF5A-1 isoform A	gi|219555707	152	97.7±12.9	37.0±7.1	+2.6
5502	leucine aminopeptidase 3	gi|165905571	274	159.7±24.6	84.6±12.7	+1.9
1724	ubiquitin activating enzyme E1	gi|35830	163	25.3±4.8	12.2±1.6	+2.1
3013	ubiquitin-conjugating enzyme E2 J1	gi|123858322	102	22.9±3.2	–	+
8025	chain B, ubiquitin-conjugating enzyme Ubch5b	gi|119389041	136	43.7±4.8	90.7±9.2	–2.1
***Carbohydrate metabolism***						
3512	aldehyde dehydrogenase 2	gi|187370719	186	53.0±11.5	26.0±4.2	+2.0
4515	α enolase	gi|4416381	78	32.7±5.3	15.1±0.9	+2.2
6708	glucuronidase, β precursor	gi|178056516	117	12.9±2.8	0	+
7208	chain A, fructose-1,6-bisphosphatase	gi|24987565	308	78.4±11.4	37.6±9.2	+2.1
7616	phosphoglucomutase-1	gi|116004023	319	16.2±2.0	8.5±1.2	+1.9
8710	phosphoenolpyruvate carboxykinase 2	gi|194038870	568	116.0±19.2	40.4±4.6	+2.9
9207	quinone oxidoreductase	gi|113205780	226	20.7±1.6	8.9±3.5	+2.3
9310	fructose-bisphosphate aldolase A	gi|156120479	252	153.2±32.0	305.7±15.9	–2.0
***mRNA metabolism***						
4501	tryptophanyl-tRNA synthetase isoform 2	gi|109084884	188	53.3±12.3	20.1±1.9	+2.6
2309	heterogeneous nuclear ribonucleoprotein F isoform 1	gi|57107167	137	6.0±1.5	11.9±0.6	–2.0
5523	heterogeneous nuclear ribonucleoprotein U	gi|55859526	98	26.1±3.4	12.3±2.1	+2.1
8017	heterogeneous nuclear ribonucleoprotein H1	gi|48145673	343	44.4±6.2	83.8±12.5	–1.9
8026	heterogeneous nuclear ribonucleoprotein A2/B1 isoform A2	gi|4504447	113	0	27.0±3.5	–
8122	heterogeneous nuclear ribonucleoprotein A2/B1 isoform 2 isoform 7	gi|73976092	116	0	40.6±5.5	–
***Amino acid metabolism***						
6005	asparaginase-like 1 protein	gi|57100467	164	132.8±27.2	56.3±17.6	+2.4
6401	L-arginine:glycine amidinotransferase	gi|194034805	272	17.9±2.9	0	+
7211	D-amino acid oxidase	gi|47522948	144	66.5±5.0	29.1±7.5	+2.3
***Fatty acid metabolism***						
4102	crystallin λ1	gi|47523096	243	145.6±25.9	50.0±10.2	+2.9
5703	glycerol-3-phosphate dehydrogenase 2	gi|62088378	132	6.8±0.7	12.4±1.4	–1.8
***Pyrimidine metabolism***						
5610	dihydropyrimidinase-related protein 2-like isoform 1	gi|194041527	484	63.9±12.1	25.3±6.1	+2.5
***Iron homeostasis***						
4006	ferritin L subunit	gi|10304378	261	78.9±9.4	35.9±7.9	+2.2
6801	aconitate hydratase	gi|115497728	376	15.4±2.2	8.1±1.6	+1.9
***Intracellular traffic***						
1006	RAB1A, member RAS oncogene family	gi|119620325	305	99.6±14.7	42.8±14.6	+2.3
1207	protein SEC13 homolog isoform 7	gi|109034613	382	43.6±4.9	75.8±11.0	–1.8
2201	pyrophosphatase 1	gi|194042750	364	25.2±1.2	52.5±7.4	–2.1
***Ion channel***						
8702	Ca-activated chloride channel regulator 1	gi|47523388	144	28.6±3.8	13.9±2.6	+2.1
***Cytoskeleton and cell mobility***						
0514	tubulin β4	gi|73987242	380	52.5±8.8	141.1±29.6	–2.7
1503	β tubulin	gi|57209813	88	6.4±1.1	20.3±4.4	–3.1
1010	β actin	gi|118136261	115	105.3±15.2	49.9±3.4	+2.1
1109	actin γ2	gi|49168516	130	41.0±5.5	19.1±1.0	+2.2
2124	α actin	gi|119612724	112	23.8±5.9	0	+
3306	actin α2	gi|4501883	449	157.5±13.1	69.0±7.8	+2.3
4302	actin α2	gi|149632150	310	65.8±8.2	31.1±4.6	+2.1
3102	F-actin-capping protein subunit β	gi|148222609	313	41.9±4.5	93.2±19.4	–2.2
2504	keratin 8	gi|227430407	372	45.3±2.8	18.6±1.8	+2.4
3418	keratin 10	gi|186629	85	12.8±1.2	5.9±1.4	+2.2
0309	keratin 17	gi|296202900	75	22.0±3.8	0	+
3412	keratin 20	gi|160011626	81	8.0±0.5	0	+
3809	filamin A isoform 5	gi|109132802	90	6.2±0.5	11.3±1.3	–1.8
***Secretory protein***						
6003	epididymal secretory protein E1	gi|28373999	338	395.9±83.2	69.1±30.4	+5.7

a Spot number consistent with those indicated in **Figure 3**.

b GI ID: Genbank identifier.

c Protein score indicating the confidence of identification.

d Expression quantity defined as the sum of optical density for each pixel of spot area (mean ± SEM, ×10^4^).

### Plasma Proteomics

Untreated pigs showed higher total protein content in plasma (p<0.05), likely reflecting a higher degree of intestinal water loss and dehydration during feeding, relative to AB pigs. Twenty-two protein spots were identified (**Figure 3**). Four proteins appeared at different positions on 2-DE gels, including complement component, haptoglobin, fabrinogen, and albumin. Descriptive information and the expression conditions of these spots are shown in **Table 2** with positions of the spots indicated in **Figure 3** **C** and **D**.

**Table 2:** Identified proteins in blood plasma with differential expression between AB-treated and untreated pigs (p<0.05).

Spot no	Protein Name	GI Id	Protein Score	Expression Level(AB)	Expression Level (Untreated)	Fold Change (AB/Untreated)
***Haptoglobin***						
1207	α1S haptoglobin	gi|164614625	364	34.5±8.8	72.3±34.2	–2.1
1209	haptoglobin	gi|189409353	181	15.1±1.9	107.4±27.4	–7.1
1217	haptoglobin	gi|47522826	310	30.6±	72.7±22.8	–2.4
2011	haptoglobin	gi|114667507	91	32.4±	292.7±68.1	–9.1
2212	haptoglobin	gi|189409353	306	±	100.0±34.3	–
3012	haptoglobin	gi|189409353	231	61.7±	325.4±83.5	–5.3
***Complement system***						
5107	complement C4	gi|38455780	151	12.8±1.3	32.7±5.3	–2.6
8512	complement C3	gi|47522844	181	2.0±0.7	5.5±1.4	–2.8
9101	complement C3	gi|47522844	100	15.4±2.0	7.5±1.0	+2.0
9507	complement C3	gi|47522844	100	18.2±3.6	34.4±2.8	–1.9
***Fibrinogen***						
8601	fibrinogen A-α-chain	gi|1304179	263	64.2±7.9	148.3±28.8	–2.3
8617	fibrinogen A-α-chain	gi|1304179	243	10.3±4.7	30.9±13.2	–3.0
8619	fibrinogen A-α-chain	gi|1304179	243	6.5±2.0	–	+
8620	fibrinogen A-α-chain	gi|1304179	243	39.0±0.5	–	+
***Albumin***						
3201	albumin	gi|833798	148	30.0±6.2	6.6±1.3	+4.6
4106	albumin	gi|833798	117	51.8±15.1	9.2±1.6	+5.6
4406	albumin	gi|833798	555	16.0±2.2	6.6±0.8	+2.4
***Others***						
1117	col1a1 protein	gi|13096810	167	37.8±6.7	6.8±2.2	+5.6
4107	ficolin 1	gi|119608546	80	77.2±7.5	–	+
5812	inter α-trypsin inhibitor heavy chain H4	gi|48374067	170	9.7±2.2	42.5±11.5	–4.4
6106	type I collagen	gi|30102	293	27.0±4.5	9.0±1.9	+3.0
6615	transferrin	gi|189232884	219	18.2±3.1	8.7±2.1	+2.1

Refer to Table 1 for the details of table head.

### Western-blot

**Figure 4:**
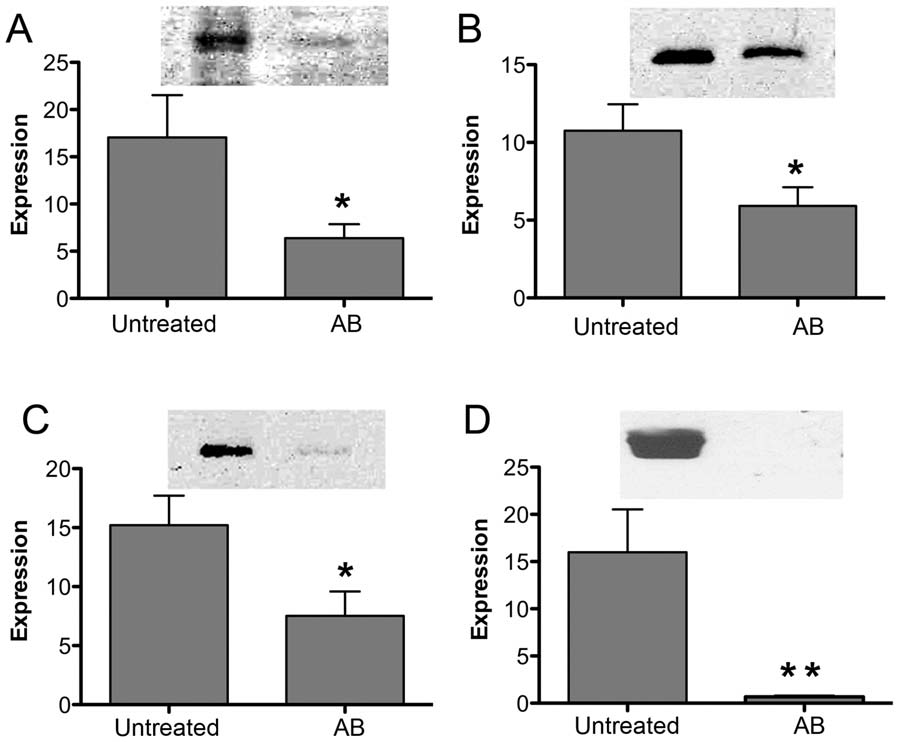
Western-blot. ***Panel A***: Laminin receptor. ***Panel B:*** pyrophosphatase 1. ***Panel C:*** HSPB1. ***Panel D:*** haptoglobin. Expression levels are presented as mean±SEM. * p<0.05 in AB *vs* untreated pigs.

The expression change of four selected proteins was further validated by Western blot (**Figure 4**). Laminin receptor (**Figure 4A**), pyrophosphatase 1 (**Figure 4B**), HSPB1 (**Figure 4C**) and haptoglobin (**Figure 4D**) all showed significantly reduced levels in the AB pigs, relative to the untreated pigs (p<0.05).

## Discussion

Treatment with systemic antibiotics is widely used for preterm infants as treatment against neonatal sepsis, or as a part of the medical intervention against NEC together with withdrawal of enteral feeding [6]–[9], [13]. However, treatment protocols vary widely among clinics and there is currently no consensus regarding the optimal neonatal antibiotics regimen to simultaneously prevent sepsis, NEC, bacterial dyscolonization and growth of antibiotics-resistant bacteria [6]–9,14,15. Antibiotics are most commonly given intravenously because intestinal immaturity and dysmotility may impair absorption of orally administered antibiotics. Regardless of delivery route, antibiotics will affect both the circulation and the gut environment, albeit in a product- and time-dependent manner [5], [9]. In this study, we chose to treat preterm pigs from birth with both intravenous and oral antibiotics to prove our hypothesis that depressed gut colonization would prevent the immature intestine from the potential damaging effects of a high density of colonizing bacteria. In our preterm pig model, we have consistently found that the sensitivity to NEC lesions increase shortly after the transition to full enteral formula feeding, concomitantly with a surge in bacterial density both in the intestinal lumen and along mucosal surfaces [10], [16]. We aimed to show benefits of reducing gut bacterial density at this critical time. Two days after the transition to full enteral feeding, we identified a large number of intestinal proteins that were differentially expressed between AB and the untreated pigs. Collectively, the nature of these proteins suggests that AB treatment supports changes in the expression of proteins related to tissue structure, function and microbial defense that may help to prevent NEC, at least short term. Further, we identified some plasma proteins that changed in response to the AB treatment and thus, could act as circulating biomarkers for the gut microbiota-related NEC development. Our follow-up studies have shown that AB-treated preterm pigs remain NEC-resistant for at least another week after completing AB treatment on day 5 after birth (NEC incidence: 0/6 vs. 9/10 and NEC score: median 1 vs. 3 in AB vs, untreated, Mann-Whitney test). Further studies are required to investigate long term effects of different early AB regimes on NEC and the developing immune system.

As the adaptive immune system is immature in preterm neonates, defense against exogenous pathogens is primarily handled by the innate immune system [17]. Complement component 3 (C3, spot 8618, **Table 1**; spot 8512, 9101, 9507, **Table 2**) that was lowered in the AB pigs, is an important constituent of the complement system, a part of innate immune system. The action of C3 is correlated with toll-like receptor (TLR) 4 and with intestinal ischemia-reperfusion [18], both of which have been suggested to play key roles in the bacteria-mediated NEC-inflammatory processes in infants and animal models [19]–[21]. C3 was also lowered in the plasma and linked with another identified protein, ficolin (spot 4107, **Table 2**). The ficolins form complexes with MBL-associated serine proteases (MASPs) in the circulation and recognize the conserved molecular patterns on the surface of pathogenic microbes. The MASP2 makes C3 convertase C4bC2b, which leads to the lysis of pathogens [17] and higher level of MASP2 is associated with more NEC in infants [22]. Further, low MBL concentration is suspected to predispose to gram-negative sepsis [17], and our findings here support this concept. There is evidence to suggest that plasma ficolin is reversely associated with the progression of NEC [23]. Besides, ficolins work together with fibrinogens (spot 8601, 8617, 8619, 8620, **Table 2**) and fibrin in the coagulation system as first line defense proteins [24]. This may explain the finding that the different isoforms of fibrinogen were significantly affected in an isoform-specific way by the AB treatment (**Table 2**).

In the plasma of the AB pigs, we found a significantly lower abundance of haptoglobin (spot 1207, 1209 1217, 2011, 2212, 3012, **Table 2**), which has been linked to inflammatory bowel disease [25]. Haptoglobin ameliorates oxidative stress [26] and participates in the intestinal innate immunology, and modulates tight junction permeability [27]. The lower level of six different isoforms of haptoglobin in the AB pigs might be due to the less strong microbial attack following reduced density of the gut microbiota. These different isoforms may possess different functions during NEC progression and the individual levels, or the total haptoglobin level, may serve as a biomarker for the microbiota-related NEC progression.

Regenerating islet-derived protein 3-γ (Reg3γ, spot 6009, **Table 1**) is a protein whose expression is triggered by increased microbial-epithelial contact at mucosal surfaces involving activation of TLR–MyD88-mediated signals in small intestine [28], [29]. The finding that the AB pigs showed lower intestinal expression of this protein corresponds with other results [30]. Similar to our findings, AB treatment with enrofloxacin and clindamycin also reduced intestinal Reg3γ mRNA and protein expression [29].

Consistent with our previous studies on germ-free preterm pigs [12], several intestinal heat shock proteins (HSPB1 (spot 4002, 4020), HSP90B1 (spot 0818), HSPA2 (spot 2720), **Table 1**) were affected by the reduced gut microbiota following AB treatment. The altered production of HSPs mitigates the injurious actions of oxidant-induced stress, probably through regulating protein homeostasis via binding, stabilizing, or refolding cell-essential proteins [31]. HSPB1 showed overall lower expression level in the AB pigs indicating decreased need for assisting the epithelial resistance to bacterial toxins and inflammation-associated stress, involving the notorious nuclear factor -κB pathway [32]. The lowered expression of HSPB1 in the completely healthy AB pigs might also relate to higher glutamine availability due to less overgrowth of bacteria and their short chain fatty acid production [31] Likewise, the AB treatment decreased HSP90B1, indicating decreased endoplasmatic riticulum stress [33], as less microbial infection induced less action of TLRs which is chaperoned by HSP90B1 [34]. Initially HSBs may respond to bacterial attack and inflammation with increased expression, while later in the progression to severe NEC lesions, the tissue stores and production of HSBs may be exhausted. This hypothesis would be consistent with our finding that HSB1 was reduced in severely NEC-affected untreated pigs, relative to germ free pigs in our earlier report [12]. Consistent with the poor enterocyte turnover in NEC pigs, the depletion of HSPA2 has been found to induce growth arrest of enterocytes from G2 to M phase and cell death via interfering with the CDC2/cyclin B1 complex [35], [36].

Bacterial growth is very iron-dependent. Correspondingly, AB treatment affected intestinal iron homeostasis via iron regulatory proteins (IRPs). IRP1 (spot 6801, **Table 1**) is converted into aconitase when the iron level is high [37]. Intestinal aconitase was elevated in our AB pigs, probably reflecting higher iron availability. Similarly, higher levels of ferritin light chain (spot 4006, **Table 1**), the major intracellular iron storage protein [37], demonstrates that intestinal tissue binds more iron for metabolism when the bacterial load is largely removed by the AB treatment. Similarly, transferrin (spot 6615, **Table 2**), a major iron transporter protein in plasma [38], was also increased in the AB pigs. Excessive iron metabolism may cause damage as this would trigger the production of reactive oxygen species (ROS) [39]. The expression of catalase (spot 7607, **Table 1**), an enzyme that is highly efficient in degrading H_2_O_2_, was increased in the AB pigs, suggesting the intestine from AB pigs may possess better capacity to cope with ROS production than untreated pigs.

Proteins related to protein synthesis, processing and degradation were also increased in the AB pigs. The identified proteins included a component of the translational machinery (laminin receptor, spot 0301, **Table 1**) [40], and also functions related to initiation of protein synthesis (eIF 5A-1 isoform A, spot 2009, **Table 1**) [41], transfer of aminoacyl-tRNA to 80S ribosomes (elongation factor 1-γ isoform 3, spot 1009, **Table 1**) [42] as well as the formation, reduction and isomerization of disulfide bonds (protein disulfide isomerase-related protein 5, spot 1402, **Table 1**) [43]. Two proteins related to the transport of newly synthesized proteins from the endoplasmatic reticulum to Golgi apparatus were identified, namely SEC13 (spot 1207, **Table 1**) [44] and RAB1A (spot 1006, **Table 1**) [45], which is also influenced by the aforementioned HSP90 [46]. The AB treatment also affected the ubiquitination system, the major system of protein degradation. The ubiquitination enzymes identified were ubiquitin-activating enzyme E1 (spot 1724), ubiquitin-conjugating enzyme E2J1 (spot 3013), and ubch5b (spot 8025). The ubiquitin-activating enzyme E1 and ubiquitin-conjugating enzymes activate the ubiquitin, and consequently transfer it to the targeted protein [47]. Multi ubiquitinated target protein is then subjected to proteolysis in the proteasome. This system is also influenced by the aforementioned protein chaperones such as HSPs and bacterial infection [47]. Consistent with changed recycling of proteins by the AB treatment, five heterogeneous nuclear ribonucleoproteins (spot 2309, 5523, 8017, 8026, 8122, **Table 1**) influencing pre-mRNA processing, modification, transport and degradation [48] were also found with changed expression.

Aminopeptidases cleave amino acids from the amino terminus of protein or peptides. In this study, activities of two aminopeptidases, ApA and ApN, were higher in AB pigs (**Figure 2E**), which is consistent with previous studies on the effects of germ-free conditions for preterm pigs after formula-feeding. [1] Another aminopeptidase, leucine aminopeptidase 3 (spot 5502, **Table 1**) which removes the NH_2_-terminal L-prolyl residues from various peptides [49], also showed higher expression in the AB pigs, again supporting the conclusion that the AB-reduced microbial load might have increased intestinal protein digestion, protein synthesis pathways and amino acid metabolism in the immature intestine.

In previous studies, NEC progression significantly affected tissue proteins associated with carbohydrate and energy metabolism [11], [12]. Most of the identified proteins in this study (7 of 9) showed higher expression level in AB versus the untreated pigs, suggesting a more active carbohydrate metabolism. Among the identified proteins, aldehyde dehydrogenase 2 transforms acetaldehyde to acetyl-CoA to enter the TCA cycle. Phosphoglucomutases 1 converts glucose-1-phosphate from a glycogen to glucose-6-phosphate, which goes into the glycolysis pathway. Aldolase A, glycerol-3-phosphate dehydrogenase 2 and enolase catalyze three different steps of glycolysis. Phosphoenolpyruvate carboxykinase 2 and glucuronidases are involved in gluconeogenesis. Inorganic pyrophosphatase plays roles in energy metabolism, provides a thermodynamic pull for many biosynthetic reactions [50].

In our previous proteomic studies on NEC, repeated identification of proteins related to cytoskeleton, cell integrity and cell mobility was a consistent observation associated with feeding and bacterial colonization [11], [12]. Similarly in this study, two tubulins, six isoforms of actin, and four keratins were identified. Actins are crucial for cellular response and healing of mucosa defects after gram-negative bacterial attack and endotoxins exposure [51]. Keratin 8 has a role also in apoptosis of colonic cells [52]. The above structural cytoskeleton proteins are also associated with aforementioned identified proteins such as C3 [53], HSPB1 and HSPA2 [31], [36].

Chloride secretion is the major driving force for intestinal fluid secretion and fluid hypersecretion induced by enterotoxin resulting in diarrhea and dehydration [54], the key features of NEC. We detected AB-induced increased abundance of Ca-activated chloride channel regulator 1 (spot 8702, Table 1) that is known to regulate the intercellular Cl^−^ efflux and trans-epithelial Cl^−^ secretion [55], [56]. Hence, the AB-reduced bacterial load may be associated with a more efficient regulation of intestinal Cl^−^ secretion.

The optimal dose, timing, route of administration and product(s) of antibiotics in neonatology remain controversial. Using a preterm pig model of NEC, we now demonstrate very consistent beneficial effects of broad-spectrum antibiotics treatment for the first five days after birth during the difficult transition from parenteral to enteral nutrition. Our proteomic analyses indicate that the AB treatment might protect the intestine by the interacting effects of intestinal complement system, HSP protection, protein synthesis and degradation and the metabolism of iron, carbohydrates, fatty acids and amino acids. We also demonstrate that circulating levels of haptoglobin, Reg3γ, cleaved C3 and ficolin are related to these tissue effects, and therefore have a potential to act as biomarkers for the feeding- and microbiota-induced progression of NEC during the difficult first weeks of life after preterm birth. Such markers need to be validated in longitudinal studies on NEC and while our results demonstrate clear benefits of early broad-spectrum antibiotics on the preterm pig intestine, the optimal dose, product and route of delivery to provide short and long term benefits in both preterm pigs and infants remain to be defined.

## Materials and Methods

### Ethics Statement

All the procedures on animals were approved by the National Committee on Animal Experimentation in Denmark (permit no. 2009/561–1731). Surgical interventions were performed under anesthesia, and pigs were immediately euthanized if they showed extensive discomfort due to procedure- or disease-related reasons.

### Animals and their Treatment

Delivery, and housing of the premature piglets were carried out as previously described [1]. Briefly, preterm piglets from two sows were delivered by caesarean section at 105–106 day (90%) gestation. All piglets were housed individually in incubators with regulated temperature, moisture, and oxygen. Immediately after birth, a vascular catheter (infant feeding tube 4F; Portex, Kent, UK) was inserted into the dorsal aorta via the umbilical cord of the anesthetized newborn pigs for parenteral nutrition, and an orogastric feeding tube (6F Portex) was provided for enteral feeding. After recovery from surgery, all piglets were given PN as well as minimal enteral nutrition (3 mL formula per 6 h) for 2 days. After that, all piglets were switched to total enteral nutrition (infant formula, 15 mL/kg/3 h) via the orogastric tube. The formula was composed of Peptide 2–0 (SHS, Liverpool, UK), 80 g, Laprodan 15 (ARLA, Aarhus, Denmark) 70 g, and 75 mL of Liquigen-MCT (SHS, Liverpool, UK) per L water, and designed to match the composition of sow’s milk during lactation.

Piglets were stratified between treatments according to sex and birth weight. Six piglets were given the antibiotic treatment daily (AB group), and six other piglets served as untreated controls and were given a corresponding volume of saline. The antibiotic treatment was 100 mg/kg per day ampicillin (Pentrexyl, Bristol-Myers Sqibb, Bromma, Sweden, distributed equally between 50 mg i.m. and 50 mg i.g. daily doses), 2.5 mg/kg per day gentamycin (given i.m. and i.g., KU-LIFE Pharmacy, Copenhagen, Denmark) and 10 mg/kg per day metronidazole (Flagyl, Sanofi Aventis, Hørsholm, Denmark and Metronidazole, Actavis, Hafnarfjordur, Island, given i.m and i.g., respectively). The product and doses were chosen based on the i.v. antibiotics regime generally used for septic infants at the Department of Neonatology, Copenhagen University Hospital (Copenhagen, Denmark).

### Tissue Collection

If clinical signs of NEC were prior to the predetermined conclusion of the experiment on day 5 after delivery, the piglets were euthanized by sodium pentobarbital (200 mg/kg, i.a.). Otherwise the piglets were euthanized and sampled on the fifth day, 45–55 h after initiation of full enteral feeding. Just before euthanasia, a blood sample was collected from the arterial catheter, and plasma was separated and stored at −20°C for further analyses. Clinical signs of NEC was recorded according to our macroscopic NEC evaluation system [16], where 1  =  absence of lesions, 2  =  local hyperemia, inflammation, and edema, 3  =  hyperemia, extensive edema, and local hemorrhage, 4  =  extensive hemorrhage, 5  =  local necrosis and pneumatosis intestinalis, and 6  =  extensive necrosis and pneumatosis intestinalis. NEC was defined as a score of minimum 3 in minimum one intestinal region (proximal, middle, distal intestine, colon).

The gastrointestinal tract was immediately removed, and a 6 cm section of the middle small intestine was saved for histological analysis, brush border enzymatic activity assays and proteomic analysis. Tissue sections for histological analysis were fixed in 4% paraformaldehyde. Samples for enzymatic activity assays and proteomic analysis were frozen in liquid nitrogen and stored at −80°C. Another 10 cm section of the mid intestine was collected for determining the proportion of intestinal mucosa [16].

### Villous Morphology, Brush-border Enzymes and Gut Microbiology

The paraformaldehyde fixed samples were embedded in paraffin and sectioned at 5 µm and stained with HE. All slides were checked with a light microscope (Orthoplane, Leitz) and imaginized by NIH Image J software (NIH, USA). Snap-frozen mid intestinal sections were homogenized in 1.0% Triton X-100 and the homogenates were assayed for activities of disaccharidases (lactase, maltase, sucrase) and peptidases (ApA, ApN, DPPIV) as described previously [57]. Densities of bacteria in luminal content obtained from the ceacum was enumerated by conventional culture-based microbiology [58]. The cultures for total aerobic and anaerobic bacteria were carried out on calf blood agar plates (SSI Diagnostika, Hillerød, Denmark). The number of colony forming units was then determined using serial dilutions, and enumerated on the highest countable dilution.

### Gel-based Proteomics

Extraction of intestinal protein, 2-DE and protein identification was carried out as previously described [11], [59]. Briefly, the tissue samples were disrupted with a tissue teaser (Biospec Products, OK, USA) in a cocktail buffer containing Triton X-100 and Protease Inhibitor Cocktail Set 3 (Bio-Rad, Hercules, CA, USA). The protein extracts were further purified with precipitation with trichloroacetic acid-acetone solution. The purified protein was dissolved in a buffer containing 7 mol/L urea, 2 mol/L thiourea, 4% Chaps, 100 mmol/L dithiothreitol, and 5% glycerol. Protein concentration of the samples was determined by Bio-Rad Protein Assay (Bio-Rad).

Intestinal protein sample (100 µg), or heparin-treated blood plasma (5 µL) was mixed with rehydration buffer (9.5 mol/L urea, 2% Chaps, 0.28% dithiothreitol, 0.5% IPG Buffer pI 3–10) and applied onto one ReadyStrip IPG Strip (18 cm, pI 3–10 NL, Bio-Rad). The isoelectric focusing was carried out on an Ettan IPGphor 3 (GE Healthcare, Uppsala, Sweden) with specific running program after an active rehydration step. The SDS-PAGE of the focused gel strips were carried on 1.0 mm-thick 12.5% PAGE gels. After electrophoresis, gels were stained with SYPRO Ruby Protein Stain (Bio-Rad) according to the manufacturer’s guide. The stained gels were scanned with a Molecular Imager PharosFX Plus System (Bio-Rad) and analyzed by PDQuest 8.0 (Bio-Rad). Matched spots were assigned with numbers automatically.

Gel spots with significant abundance between the AB and untreated pigs (p<0.05) were manually cut out and applied to in-gel trypsin digestion. The peptide mixtures obtained were applied on a 4800 MALDI TOF/TOF Analyzer (Applied Biosystem, Carlsbad, CA, USA) for MALDI-TOF/TOF MS. Generated mass spectrum was used for the protein identity searching with searching taxonomy limited as *Mammalia* (mammals) against NCBInr database. A protein match with a protein score > 71 was considered significant.

### Western-blot Analysis

Briefly, 20 µg intestinal protein or 1 µL plasma was resolved by electrophoresis on a 12.5% SDS-PAGE gel. The expression of laminin receptor, pyrophosphatase 1, HSPB1 and haptoglobin were shown with specific antibody (ab65436, ab96099, ab12351, ab14248, Abcam, Cambridge, UK). The protein bands were visualized and the density of the protein bands was detected by Quantity One (Bio-Rad).

### Statistical Analysis

The discrete NEC scores of the treatment groups were reported as medians, and the difference between groups evaluated with non-parametric Mann-Whitney test using GraphPad Prism 5 (GraphPad software, La Jolla, CA, USA). The abundance of proteins by proteomics and Western blot analysis were expressed as mean ± SEM and analyzed with two-tailed Student *t* test with Levene’s Test for equality of variances in SPSS 11.5. A p value less than 0.05 was considered significant.
